# Metformin Prevents Nigrostriatal Dopamine Degeneration Independent of AMPK Activation in Dopamine Neurons

**DOI:** 10.1371/journal.pone.0159381

**Published:** 2016-07-28

**Authors:** Jacqueline A. Bayliss, Moyra B. Lemus, Vanessa V. Santos, Minh Deo, Jeffrey S. Davies, Bruce E. Kemp, John D. Elsworth, Zane B. Andrews

**Affiliations:** 1 Department of Physiology, School of Biomedical and Psychological Sciences, Monash University, Clayton, Melbourne, Vic., 3800, Australia; 2 Molecular Neurobiology, Institute of Life Science, Swansea University, Swansea, SA28PP, United Kingdom; 3 St Vincent’s Institute & Department of Medicine, The University of Melbourne, 41 Victoria Parade, Fitzroy, Victoria 3065, Australia; 4 Department of Psychiatry, Yale University School of Medicine, New Haven, Connecticut 06520, United States of America; CRCHUM-Montreal Diabetes Research Center, CANADA

## Abstract

Metformin is a widely prescribed drug used to treat type-2 diabetes, although recent studies show it has wide ranging effects to treat other diseases. Animal and retrospective human studies indicate that Metformin treatment is neuroprotective in Parkinson’s Disease (PD), although the neuroprotective mechanism is unknown, numerous studies suggest the beneficial effects on glucose homeostasis may be through AMPK activation. In this study we tested whether or not AMPK activation in dopamine neurons was required for the neuroprotective effects of Metformin in PD. We generated transgenic mice in which AMPK activity in dopamine neurons was ablated by removing AMPK beta 1 and beta 2 subunits from dopamine transporter expressing neurons. These AMPK WT and KO mice were then chronically exposed to Metformin in the drinking water then exposed to MPTP, the mouse model of PD. Chronic Metformin treatment significantly attenuated the MPTP-induced loss of Tyrosine Hydroxylase (TH) neuronal number and volume and TH protein concentration in the nigrostriatal pathway. Additionally, Metformin treatment prevented the MPTP-induced elevation of the DOPAC:DA ratio regardless of genotype. Metformin also prevented MPTP induced gliosis in the Substantia Nigra. These neuroprotective actions were independent of genotype and occurred in both AMPK WT and AMPK KO mice. Overall, our studies suggest that Metformin’s neuroprotective effects are not due to AMPK activation in dopaminergic neurons and that more research is required to determine how metformin acts to restrict the development of PD.

## Introduction

Parkinson’s Disease (PD) is the second most common neurodegenerative disease affecting an estimated 4.1 to 4.6 million people worldwide in 2005, a number projected to double by the year 2030 [1]. Symptoms including tremor, postural instability and bradykinesia are due to a reduction of dopamine in the dopaminergic nigrostriatal pathway in the brain. The dopamine cell bodies are located in the substantia nigra (SN) and project to the striatum. PD is considered idiopathic however many risk factors occur to increase incidence rates such as genetic factors, pesticide exposure and recently several studies have shown a greater incidence in patients who have diabetes [2–4]. Intriguingly, there is a correlation between the incidence of diabetes preceding PD development in individuals [5], indicating that glucose intolerance may be a precipitating factor in the development of PD. Indeed, this is true in other neurological diseases where individuals with Type 2 Diabetes (T2D) are at risk of developing mild cognitive impairment, dementia or Alzheimers [6, 7]. If glucose intolerance is an early event or precipitating factor in neurological conditions, then current therapeutic approaches to treat diabetes may offer insights into the pathogenesis of neurological disease, such as PD. In support of this concept, retrospective epidemiological study showed that Metformin-inclusive sulfonylurea therapy reduced the risk of PD occurrence in patients with T2D in a Taiwanese population [8].

Metformin is a buigiunide analogue commonly used for the treatment of T2D and is generally well tolerated. By lowering blood glucose, IGF-1 and insulin signalling, Metformin creates an environment that is similar to calorie restriction (CR) and as such many beneficial effects of CR can be reproduced by chronic Metformin treatment. Metformin has been shown to extend median survival by 40% in C. elegans, whilst also prolonging youthful locomotion in a dose-dependent manner [9]. In mice Metformin produced approximately a 6% lifespan extension, which was also accompanied by improved locomotor performance [10]. Indeed, in a human study patients with T2D with Metformin monotherapy had a longer survival than matched non-diabetic controls [11]. These studies collectively imply not only enhanced lifespan but also healthspan with Metformin treatment. Metformin treatment also reduces the incidence of many age related diseases by reducing cancer incidence [12], stroke risk [13], enhancing neurogenesis [14] as well as the traditional lowering of blood glucose. As CR is beneficial for PD [15] and T2D [16], Metformin has the potential to treat both disease states.

Previous studies show that Metformin is neuroprotective in PD. In vitro, treatment with Metformin reduced the neurotoxicity associated with alpha synuclein overexpression [17]. In a Drosophila model of PD, Metformin treatment alleviated dopaminergic dysfunction and mitochondrial abnormalities [18]. Metformin chronically administered to mice reduces oxidative stress, dopaminergic degeneration and motor abnormalities associated with MPTP (a mouse model for PD) administration [19]. Hence, Metformin treatment has a protective effect in PD. As Metformin has been deemed safe with minimal side effects and is known to rapidly cross the blood brain barrier and disperse into various brain regions [20], it is an ideal therapeutic for the treatment of PD. Recently, a mechanism of action for how Metformin suppresses gluconeogenesis has been discovered [21], however, how Metformin is neuroprotective is still unknown. For example, after Metformin treatment the transcription factor SKN/Nrf2 is activated, ultimately increasing the expression of anti-oxidant genes to protect against oxidative damage [9]. Metformin has also been shown to inhibit mTOR to enhance mitochondrial function [22, 23]. Metformin can also activate AMPK by inhibiting complex I of the ETC [24]. This results in an increased AMP/ATP ratio and the subsequent activation of AMPK. AMPK acts to increase mitochondrial biogenesis [25] and as patients with PD have impaired mitochondrial function, suggesting that AMPK activation in dopamine neurons may be responsible for Metformin’s protective actions.

Peripheral activation of AMPK has been shown to be protective in PD. In cells overexpressing alpha synuclein AMPK becomes activated to restrict cell death [17]. In a Drosophila model of PD, loss of AMPK activity exacerbated neuronal loss and its associated phenotypes [18]. Resveratrol, an AMPK activator is neuroprotective in a rodent model of PD [26]. In response to MPTP AMPK is phosphorylated and inhibition of AMPK by Compound C enhanced MPTP-induced cell death [27]. However, it is unknown whether AMPK activation in dopaminergic neurons is causative or correlative in the pathogenesis of PD. Many studies have highlighted the beneficial effects of peripheral AMPK activators in PD [26, 28, 29] however, these neuroprotective actions could be due to indirect actions and may not involve AMPK directly in dopamine neurons or could be due to an overall enhanced peripheral profile.

As both Metformin treatment and AMPK activation protect against PD, we hypothesised that Metformin would activate AMPK in dopaminergic neurons to prevent degeneration. We aimed to determine if Metformin’s neuroprotective effects can be attributed to the activation of AMPK in the SN dopamine neurons.

## Material and Methods

### Animals

All experiments were conducted in accordance with Monash University Animal Ethics Committee guidelines. The experiments in the study were specifically approved the Monash Animal Research Platform Animal Ethic Committee (MARP/2012/042). Male mice were maintained under standard laboratory conditions with free access to standard chow and water. Environmental enrichment consisting of tissues and cardboard boxes cut to fit the cages was provided in every cage. Mice were weighed and monitored daily. No mice died prior to the endpoint. Due to the use of MPTP early termination was designated if suffering was observed or if body weight fell below 70% of initial weight. All efforts were made to minimise suffering and isoflurane was used at end of experiment. Temperature was maintained at 23°C with a 12 hour light/dark cycle. Mice were 8–10 weeks old and group housed.

To generate mice with a selective deletion for AMPKβ1 & β2 only in DAT-expressing neurons, we crossed Dat-Cre knock-in mice from Jax Lab Lab [Stock number 006660; B6.SJL-Slc6a3<tm1.1(cre)bkmn>/j] with AMPK beta 1 (β1) and beta 2 (β2) floxed mice [30]. The resultant offspring (*Dat*-Cre;*Ampk beta 1*^*fl/fl*^;*Ampk beta 2*^*fl/fl*^ designated AMPK KO or *Ampk beta 1*^*fl/fl*^;*Ampk beta 2*^*fl/fl*^ designated AMPK WT) were used as experimental mice. Experimental validation of mice was performed previously [31].

### Experimental Design

Mice were randomly allocated to be treated with water or water containing Metformin (100mg/kg/day) for the duration of the experiment (27 days). Metformin was given in water to maintain constant levels of Metformin in the bloodstream and sustained activation of AMPK as opposed to a single dose which is cleared rapidly from the system. Previous literature in stroke models using 100mg/kg/day of Metformin showed increased AMPK activation in the brain with this dose [32]. On days 20 and 21 mice received an injection of either saline or 1-methyl-4-phenyl-1,2,3,6-tetrahydropyridine (MPTP) (30mg/kg dissolved in saline). Mice were deeply anaesthetised using isoflurane and were culled on day 27 and either perfused for immunohistochemical analysis or fresh tissue collection for western blot and HPLC analysis.

### Immunohistochemistry

All mice were deeply anaesthetized with isoflurane then perfused with 0.05M PBS followed by 4% Paraformaldehyde (PFA) to fix the tissue. Brains were stored in PFA overnight then transferred to 30% sucrose solution. Every fifth coronal section (30μm) was collected and stored in cryoprotectant (30% Ethylene Glycol, 20% Glycerol in 0.1M PB) at -20°C. The sections were washed thoroughly with 0.1M PB and endogenous peroxidase activity was blocked with 1% H_2_O_2_ in 0.1M PB for 15 minutes and washed again. The tissue was blocked with 4% normal horse serum and 0.3% Triton X-100 in 0.1M PB for one hour and then blocked again with AffiniPure Goat Anti-Mouse IgG (H+L) (Jackson ImmunoResearch, 1:200) to prevent non-specific binding due to mouse tissue being stained with mouse antibodies. The tissue was then incubated with the primary antibody, in this case anti-TH (mouse, 1:5000, Milipore) and anti-IBA1 (rabbit, 1:1000, Wako) or anti-GFAP (rabbit, 1:1000, DAKO) for 24 hours at 4°C. After the incubation the sections were thoroughly washed and incubated with the fluorescent secondary antibodies Goat anti-mouse IgG (H+L) AlexaFluor 488 (1:400, Invitrogen) and Goat anti-rabbit IgG (H+L) AlexaFluor 594 (1:400, Invitrogen) for 90 minutes at room temperature. The tissue was subsequently washed, mounted onto slides and cover-slipped using anti-fade mounting media.

### Stereological analysis of cell number and volume

We used design-based stereology to quantify the number of microglia (IBA1 stain), astrocytes (GFAP stain) and the number and volume of dopamine neurons (TH stain) in the Substantia Nigra. Using the StereoInvestigator software (MicroBrightField, Williston, VT, USA) we analysed cell number (optical fractionator probe) and cell volume (nucleator probe). To visualise the cells we used a Zeiss microscope with a motorised stage and a MicroFibre digital camera connected to the computer.

### Analysis of blood chemistry

Trunk blood from deeply anesthetised decapitated mice was collected into EDTA tubes pre-treated with pefabloc (SC Roche Applied Science, Mannheim, Germany) to achieve a concentration of 1mg/mL. The blood was centrifuged and the plasma was acidified with HCl (final concentration 0.05N). Plasma metabolites were measured following kit instructions. Active Ghrelin or Des-Acyl Ghrelin Enzyme-Linked Immunoassay Kits (Mitsubishi Chemical Medicine, Tokyo, Japan), NEFA (Wako Life Sciences; CA, USA), Triglycerides (Roche/Hitachi; Indianopolis, USA), Corticosterone (Abnova; CA, USA) and Blood glucose (Sigma; Missouri, USA). Plasma insulin concentration was determine through an in-house ELISA assay.

### Western Blot

Fresh tissue was collected of the SN and Striatum and snap frozen in liquid nitrogen. Tissue was then sonicated in RIPA buffer (50mM Tris, 150mM NaCl, 0.1% SDS, 0.5% sodium deoxycholate, 1% Triton X-100) containing a protease inhibitor (Sigma), then centrifuged (10,000 rpm, 10 minutes, 4°C) to remove cell debris and the supernatant was collected. The protein concentration was measured using a BCA kit (Pierce, Rockford, IL, USA) according to kit instructions. The samples concentrations were then standardised and the supernatants were mixed with Laemmli’s buffer and boiled for 5 minutes. Samples were loaded onto 10% acrylamide gels and separated by SDS polyacrylamide gel electrophoresis, the separated proteins were then transferred from the gel to a PVDF membrane (Biorad). The blots were bocked for 1 hour in Tris-Buffered Saline Solution containing 0.1% Tween-20 (TBST) and 5% Bovine Serum Albumin (BSA). The membrane were incubated overnight at 4°C in TBST with 5% BSA containing one of the following antibodies: anti-TH (1:1000, Milipore), anti-beta actin (1:1000, Abcam), anti-AMPKα (1:1000, Cell signalling) or anti-pAMPK (1:1000, Cell Signaling). Blots were visualised using chemiluminescence (ECL, Amersham) and levels of proteins were detected using ImageLab Software, version 4.1, Biorad.

### High Performance Liquid Chromatography (HPLC)

We performed HPLC to identify, separate and quantify both dopamine and DOPAC concentrations within the striatum. Striatal tissue was rapidly dissected and snap frozen (approximately -70°C). The samples were then sonicated in 0.4mL cold 0.1M perchloric acid containing the internal standard. After centrifugation DA, DOPAC and the internal standard in the supernatant were extracted on alumina at pH 8.4, eluted in 0.1M perchloric acid, separated by reverse-phase HPLC and detected using electrochemical detection. The concentration of dopamine and DOPAC were calculated in reference to the internal and external standards. The Lowry method was used to determine the protein content of each sample from the centrifuged pellet. The concentrations of dopamine and DOPAC are expressed as ng/mg of protein present (mean ± SEM).

### Oral Glucose Tolerance Test (oGTT) and Insulin Tolerance Test (ITT)

Mice were fasted for 4 hours prior to first blood measurement. We used short term fasting as this better studies insulin action in the physiological context [33]. Blood glucose was measured with ACCU-CHEK Active (Roche DiagnosticsGnH, Tokyo, Japan) and then a 25% D-glucose solution (1.5g/kg) was given by oral gavage and measurements were taken at time points 15, 30, 60 and 90 minutes after bolus. The mice were allowed to recover for 1 week until the ITT was performed. For the ITT the same protocol was followed except the mice were injected i.p 1U/kg insulin.

### Statistical Analysis

All data are represented as Mean ± Standard Error of the Mean (SEM). Two-Way ANOVA with a Bonferroni post hoc test was used to determine statistical significance between treatments. P<0.05 was considered statistically significant.

## Results

### Physiological characterisation of AMPK KO mice

**Fig 1:**
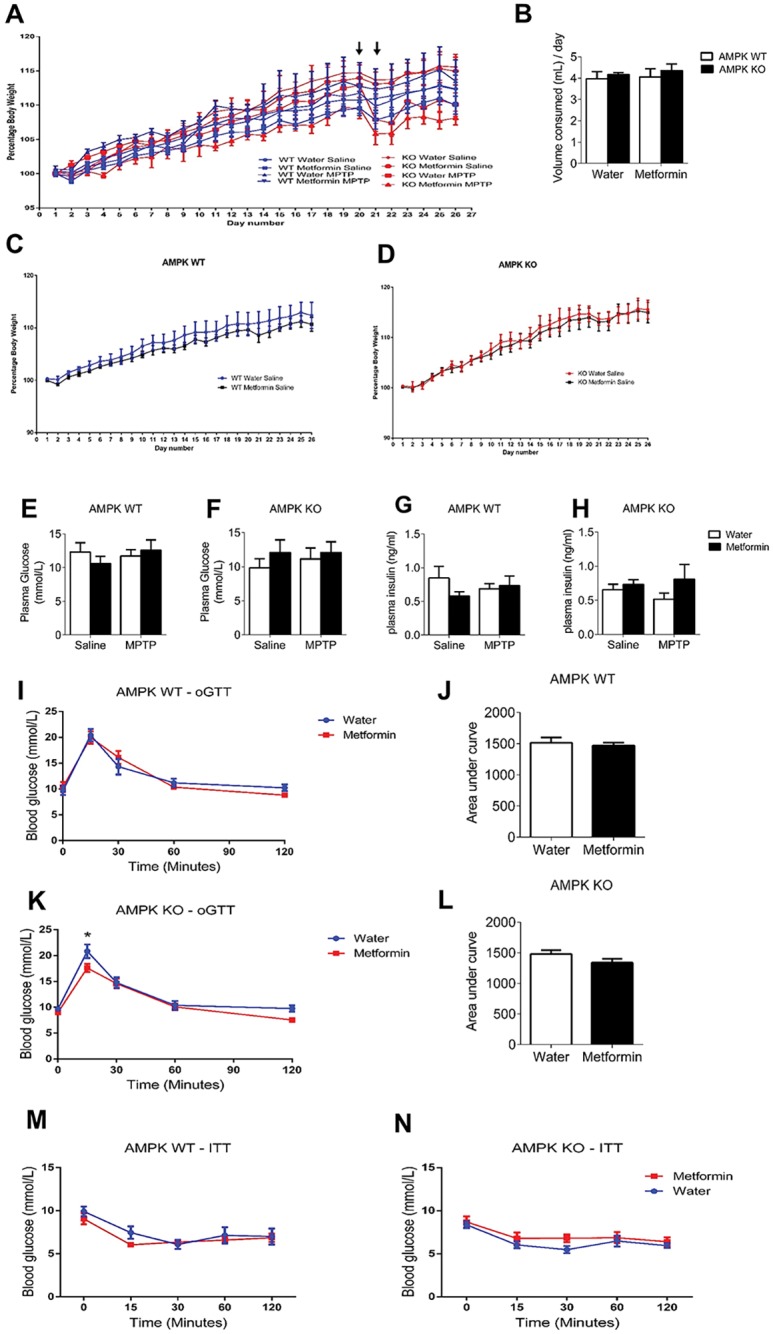
Body Weight and blood glucose measurements in AMPK WT and KO mice. **A–D**, throughout the experiment there was no difference in body weight or volume of water consumed comparing genotype or treatment. Arrows indicate MPTP injections. **E–H**, Plasma analysis of insulin and glucose levels in trunk blood show no differences between genotype and treatment. During an oGTT Metformin treatment does not alter glucose clearance in AMPK WT (**I & J**) or AMPK KO mice (**K & L)**. Insulin sensitivity is not altered during an ITT in AMPK WT (**M**) or KO (**N**) mice treated with Metformin. * = p<0.05. Data are represented as mean ± SEM (n = 8–10, two-way ANOVA, p<0.05).

DAT AMPK WT and KO were exposed to either normal drinking water or water with Metformin and then injected with MPTP to induce degeneration in Substantia Nigra (SN) dopamine neurons, as a mouse model for PD (raw data available; S1–S11 Files). There were no genotype differences in body weight (Fig 1A, 1C & 1D), blood glucose (Fig 1E & 1F) or insulin levels (Fig 1G & 1H). Because Metformin is known to promote insulin sensitivity [34] we determined if Metformin in the drinking water affected clearance of glucose during an oral Glucose Tolerance Test (oGTT) and Insulin Tolerance Test (ITT). In AMPK WT mice there was no difference between mice chronically exposed to Metformin compared to tap water alone in both oGTT (Fig 1I), Area under the curve (Fig 1J) and the ITT (Fig 1M). However, in AMPK KO mice Metformin significantly increased glucose clearance (Fig 1K) at the 15-minute time-point, however the area under the curve showed no significant differences between water and Metformin treatment (Fig 1L). There was no difference in the ITT (Fig 1N). This indicates that AMPK activation in dopamine neurons does not promote peripheral glucose clearance during an oGTT or ITT.

**Fig 2:**
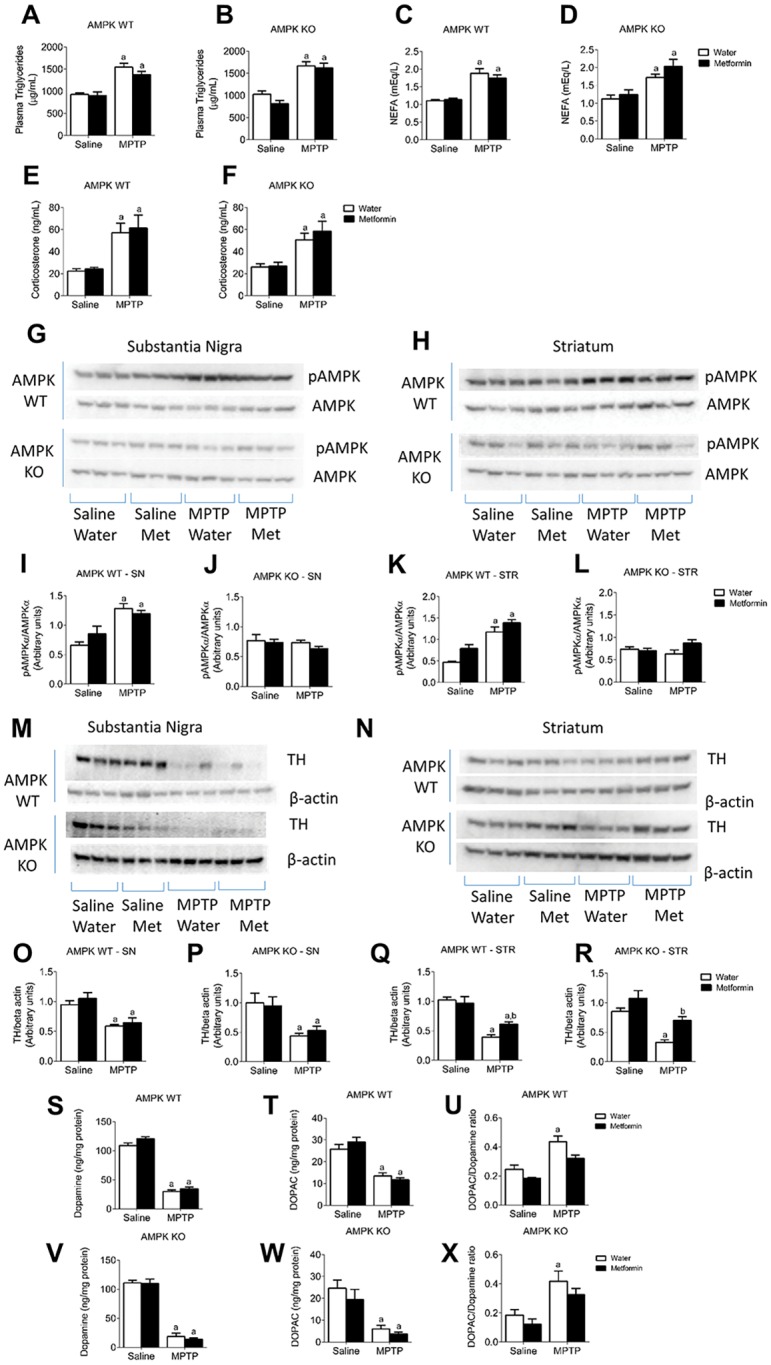
Metformin is neuroprotective in AMPK WT and KO mice. Plasma Triglycerides (**A & B**), NEFA (**C & D**) and corticosterone (**E & F**) are elevated in response to MPTP. Representative Western Blot images of pAMPK/AMPK in the SN (**G**) and Striatum (**H**). Protein analysis of the pAMPK/AMPK ratio showing no elevation in response to MPTP in the SN (**J**) and Striatum (**L**) in AMPK KO but an elevation in both the SN and Striatum of AMPK WT mice (**I & K**). Representative images of TH levels in the SN (**M**) and Striatum (**N**). In the SN there is a significant reduction in TH levels in both AMPK WT (**O**) and KO (**P**) in response to MPTP. In the Striatum Metformin elicits a neuroprotective effect in MPTP treated AMPK WT (**Q**) and KO (**R**) mice. MPTP reduced dopamine and DOPAC in both AMPK WT (**S & T**) and AMPK KO (**V & W**) mice. Metformin reduced the elevation of the DOPAC:DA ratio in MPTP treated mice compared to water alone, in AMPK WT and KO mice (**U & X**) a, significant compared to water/saline treated mice and b, significant compared to water/MPTP treated mice. Data are represented as mean ± SEM (n = 6–9, two-way ANOVA, p<0.05).

Plasma analysis showed a significant main effect of MPTP to increase Triglycerides (Fig 2A & 2B), Non-Esterified Fatty Acids (NEFA) (Fig 2C & 2D) and corticosterone (Fig 2E & 2F) levels, which occurred with a concurrent reduction in body weight (Fig 1A) indicating the stress placed on these mice. These hormones were measured to determine if metabolic feedback was playing a role in the neuroprotective actions of Metformin. To confirm the inability of AMPK to become phosphorylated in the AMPK KO mice we performed protein analysis in the SN and Striatum. In both the SN and striatum AMPK WT mice had an elevated pAMPK/AMPK ratio after MPTP exposure (Fig 2I & 2K). AMPK KO mice exhibited no significant change in the pAMPK/AMPK ratio in response to MPTP in either the SN (Fig 2J) or Striatum (Fig 2L) implying an inability to be phosphorylated in response to a toxic insult as a protective mechanism, as observed previously [31].

### Metformin prevents MPTP-induced nigrostriatal damage independent of genotype

MPTP significantly reduced Tyrosine Hydroxylase (TH, a dopamine marker) protein expression, as measured by western blot in both the SN and Striatum in both AMPK WT and KO mice (Fig 2M–2R) an effect that was attenuated by metformin in both AMPK WT and KO mice in the Striatum (Fig 2Q & 2R) but not in the SN (Fig 2O & 2P). In support of this data, HPLC analysis of dopamine (Fig 2S & 2V) and DOPAC (Fig 2T & 2W) in the striatum revealed a significant overall reduction with MPTP administration in both AMPK WT and KO mice with no effect of Metformin treatment. However, Metformin treatment prevented the increase in the DOPAC:DA ratio observed after MPTP in both AMPK WT and KO mice (Fig 2U & 2X). These results indicate that Metformin has site-specific protective effects in the Striatum.

**Fig 3:**
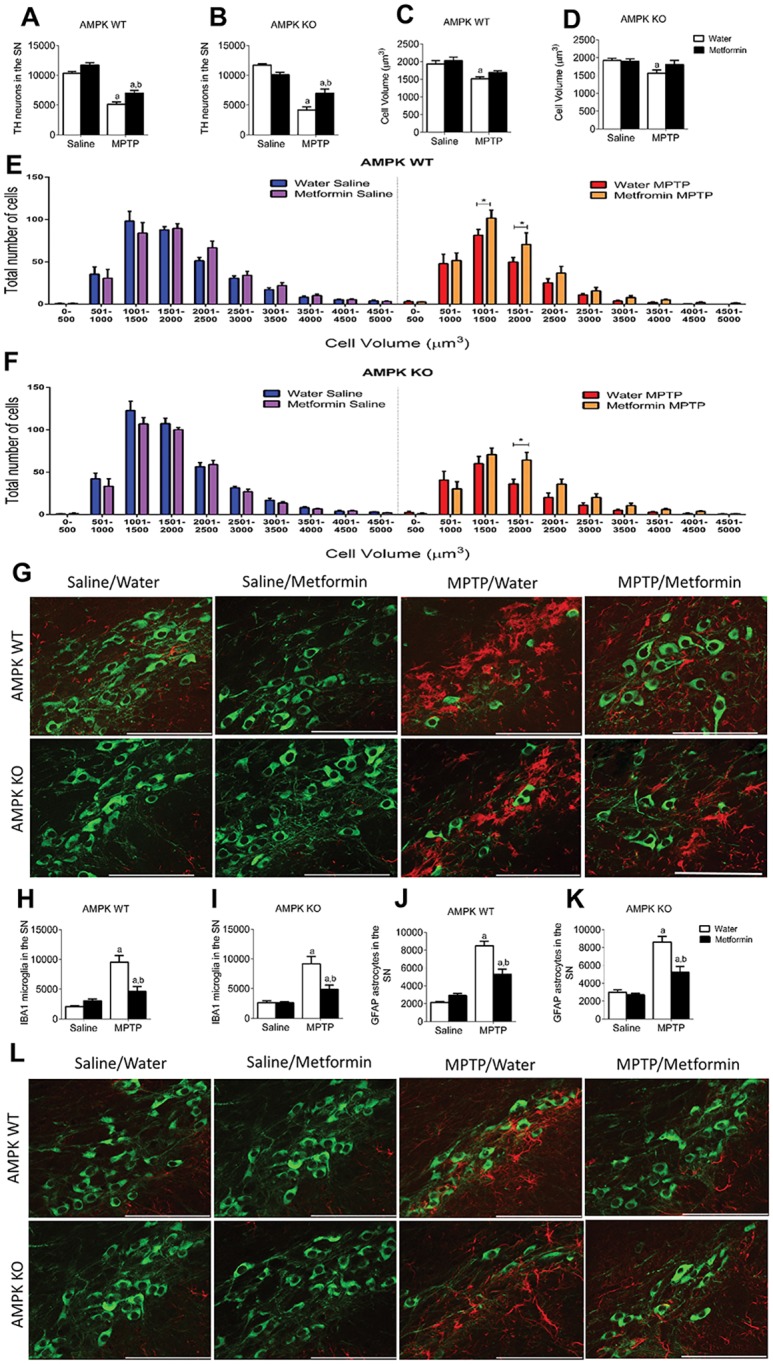
Metformin preserves cell number and volume after MPTP exposure, independent of genotype. Stereological quantification of TH levels in the SN shows a protective effect of Metformin after MPTP exposure in both AMPK WT (**A**) and KO (**B**) mice. Overall cell volume shows a significant reduction after MPTP exposure in water treatment but not with Metformin treatment in AMPK WT (**C**) and KO (**D**). When TH cells were separated and plotted based on volume distribution, mice treated with MPTP and Metformin had a significant effect on smaller volume (1000–2000μm^3^) cells compared to those not treated with Metformin in both AMPK WT (**E**) and KO (**F**) mice. **G**, Representative image showing MPTP induced microglial activation in the SN (green = TH, red = IBA1). Stereological quantification of IBA1 (**H & I**) and GFAP (**J & K**) showing increased numbers after MPTP with a significant protective effect of Metformin in both AMPK WT and KO. **L**, Representative images showing MPTP induced astrocytic activation in the SN (green = TH and red = GFAP). a, significant compared to Water/saline treated mice and b, significant compared to Water/MPTP treated mice. * = p<0.05. Data are represented as mean ± SEM (n = 7–10, two-way ANOVA, p<0.05).

MPTP administration significantly reduced the number and volume of TH positive SN neurons in both AMPK WT and KO mice. Metformin attenuated the loss of neurons in AMPK WT (Fig 3A) and KO mice (Fig 3B) and also prevented a reduction in cell volume in both genotypes (Fig 3C & 3D), with the majority of the beneficial effect occurring in smaller cells between 1000–2000μm^3^ (Fig 3E & 3F). This protective effect was also accompanied by reduced gliosis. MPTP administration significantly elevated microglia (IBA1+ cells) and astrocytes (GFAP+ cells) in the SN, which indicates greater cellular damage in that region. MPTP significantly elevated IBA1 (Fig 3G–3I) and GFAP (Fig 3J–3L) in both AMPK WT and KO mice. Metformin treatment blunted the gliosis response to MPTP in both AMPK WT and KO mice, indicating a protective effect of Metformin regardless of genotype (Fig 3H–3K).

Together, the TH cell number, cell volume and protein expression in the Striatum data indicate that Metformin elicits a protective effect in an MPTP mouse model of PD independent of AMPK action in the dopaminergic neurons.

## Discussion

Metformin has been a major therapeutic option for the treatment of T2D since 1958 in the UK and 1995 in the USA. It is the most commonly prescribed drug in the treatment of T2D in clinical use. Recently, the use of Metformin to treat diseases other than T2D has increased. Metformin prevents the development of renal [35], hepatic [36], cardiac [37, 38] and neurological conditions [39]. Indeed, Alzheimers Disease (AD) is closely associated with impaired insulin signalling and glucose metabolism and is often referred to as “type 3 diabetes” [40]. There is a correlation between insulin resistance and an elevated risk for AD development [41] and studies conducted in obese leptin-resistant mice showed that Metformin attenuated AD-like neuropathology and biological markers [39]. These studies collectively imply that Metformin has the ability to negate any elevated risk for AD development in the susceptible patients who already have T2D. However, it should be noted in a population based study following >7000 people taking Metformin (or other antidiabetic drugs) there was no elevated risk for developing AD [42].

Metformin has recently been shown to have neuroprotective effects in PD patients. A recent epidemiological study illustrated that the incident Parkinson’s risk in patients with pre-existing diabetes treated with sulfonylureas increased by 57%, which was prevented when co-medicated with Metformin [8]. We show that Metformin treatment is neuroprotective by attenuating dopamine number and volume loss, reducing gliosis, restricting TH protein loss and enhancing dopamine turnover in the striatum. This study highlights the neuroprotective potential of Metformin to reduce the risk for developing PD and although the evidence for neuroprotection is clear in animal and cell-based models [19, 43, 44], the mechanism underlying this effect is not.

One of Metformin’s mechanisms of action in terms of insulin sensitivity is to promote AMPK activation as shown in cells [24] and tissues such as the liver [24] and muscle [45]. In our studies we clearly demonstrate that Metformin reduces degeneration of the nigrostriatal system in a mouse model of PD, however these protective effects are not dependent on AMPK activation in dopamine neurons. In this study we used a mouse model where AMPKβ1 and AMPKβ2 were successfully deleted in DAT expressing neurons. AMPK is composed of three subunits: alpha, beta and gamma. The alpha subunit plays a catalytic role whereas beta and gamma are regulatory. We chose the beta subunit as deletion of both AMPKβ1 and AMPKβ2 in muscle ablated AMPK phosphorylation [30]. A potential limitation in this model is the use of transgenic mice. There is potential that these controls (CRE negative, floxed positive) react differently to Metformin and/or MPTP as the floxed allele has been carried from conception. We recently used this novel mouse line to show that ghrelin increases AMPK in dopamine neurons, which is responsible for the neuroprotective actions of calorie restriction in PD [31]. These results and the impaired striatal AMPK phosphorylation in AMPK KO mice in this study highlights the validity of using this mouse model to explore whether Metformin requires AMPK activation to prevent degeneration in a mouse model of PD.

AMPK activation attenuates dopaminergic dysfunction in a drosophila model of PD [18]. Other activators of AMPK including Resveratrol [26] and ghrelin [31, 46] are neuroprotective in vivo. Overexpression of alpha synuclein in cells (as a model of PD) activates AMPK in order to restrict cell death [17]. Although AMPK activation is neuroprotective in PD and Metformin induces direct protective effects through AMPK in other disease states such stroke [47], our studies show that Metformin doesn’t activate AMPK in dopamine neurons to prevent degeneration in a mouse model of PD. However, this model was using mice which selectively had AMPK activity removed in dopaminergic neurons hence AMPK could elicit neuroprotective actions within cells external to the neurons. As there was a significant reduction in gliosis with Metformin treatment in both AMPK WT and KO mice after MPTP treatment there is potential for AMPK activity in microglia / astrocytes to elicit neuroprotective actions. Indeed, in vitro studies indicate that AMPK activation within microglia suppresses pro-inflammatory responses [48]. As inflammation is a key hallmark in PD [49], AMPK activation within microglia may be responsible for the neuroprotective actions of Metformin, although this theory requires experimental proof.

There are many other potential mechanisms through which Metformin can act. For example Metformin inhibits apoptosis in neuronal cortical cells [43], prevents oxidative stress-related cellular death [50] and plays an inhibitory role on inflammatory transcription factor NF-kB [51]. In mice exposed to Metformin there was reduced superoxide leakage in the mitochondria, indicating greater efficiency of mitochondrial complexes [10]. As complex I activity is diminished in PD patients [52] this enhanced mitochondrial efficiency coupled with reduced oxidative stress could be responsible for the neuroprotective actions of Metformin. Metformin also activates Sirtuins (SIRTs) and PGC-1α. SIRTs are responsible for a variety of cellular processes including enhancing mitochondrial function, cellular metabolism, gluconeogenesis as well as aging [53]. There are increased levels and activity of SIRTs in the livers of Metformin treated mice [54]. SIRT activation improves mitochondrial function and extends lifespan [55]. Indeed, lifespan is increased in mice overexpressing SIRT1 [56] and decreased in SIRT1 KO [57]. Another potential target that Metformin could act through is PGC-1α, a transcriptional regulator involved in mitochondrial biogenesis. Metformin has been shown to increase PGC-1α protein expression in the liver [58] and skeletal muscle [59]. PGC-1α deficient mice are more susceptible to MPTP-induced dopaminergic neuronal loss [60]. In mice, overexpression of PARIS, which represses the expression of PGC-1α, results in selective degeneration of substantia nigra dopaminergic neurons [61]. In humans polymorphisms of PGC-1α are associated with early onset PD [62]. Collectively, these studies show that Metformin’s neuroprotective actions could be due to many other agents that enhance mitochondrial function independent of AMPK activation. It is important to note that AMPK interacts with SIRT1 and PGC-1α. AMPK enhances SIRT1 activity, enhances the downstream target PGC-1α and increases mitochondrial biogenesis [25]. Also, AMPK can both activate and be activated by SIRT1 [63], creating a complex interplay between AMPK, SIRT1 and PGC-1α. Future research should determine if SIRT1 and PGC-1α play a role in the neuroprotective actions of Metformin.

Metformin can increase circulating levels of the gut hormone GLP-1 to help control blood glucose levels [64]. GLP1 also has receptors on SN dopaminergic neurons and prevents neurodegeneration in a mouse model of PD [65, 66]. This elevation could be responsible for the protective actions of Metformin and raises the possibility that the neuroprotective actions of metformin are secondary to changes in peripheral metabolism.

Many studies have shown the protective actions of Metformin in different disease states however, the neuroprotective mechanism of Metformin in PD remains unknown. It potentially exhibits disease and dose specific actions in different tissues. Although it is possible that Metformin is neuroprotective in other disease states such as stroke and Alzheimer’s Disease via the actions of AMPK, we show that in a mouse model of PD Metformin’s neuroprotective actions are independent to AMPK activation in dopaminergic neurons. Further research is required to determine the exact neuroprotective mechanism of action of Metformin however, some potential options involve indirect effects on metabolism including elevated GLP-1 secretion or direct effects of SIRT1 and PGC-1α in SN dopamine neurons.

## Supporting Information

S1 File Data for Corticiosterone assay. (XLSX)

S2 File Data for oral glucose tolerance tests. (XLSX)

S3 File Data for HPLC analysis. (XLSX)

S4 File Body weight data for 1 cohort of metformin treated mice. (XLSX)

S5 File Data for blood glucose. (XLSX)

S6 File Data for body weight for another cohort of mice. (XLSX)

S7 File Data for plasma insulin assay. (XLS)

S8 File Data for stereology. (XLSX)

S9 File Data for western blot analysis. (XLSX)

S10 File Data for plasma NEFA analysis. (XLSX)

S11 File Data for plasma TG analysis. (XLSX)

## Abbreviations

DA: Dopamine
GFAP: Glial Fibrillary Acidic Protein
GLP1: Glucagon Like Peptide 1
IBA1: Ionized Calcium Binding Adaptor Molecule 1
Met: Metformin
MPTP: 1-methyl-4-phenyl-1,2,3,6-tetrahydropyridine
NEFA: Non-esterified Fatty Acid
PD: Parkinson’s Disease
PFA: Paraformaldehyde
SN: Substantia Nigra
STR: Striatum
TH: Tyrosine Hydroxylase
